# Gold Nanoparticles in Single-Cell Analysis for Surface Enhanced Raman Scattering

**DOI:** 10.3390/molecules21121617

**Published:** 2016-11-25

**Authors:** Mine Altunbek, Gamze Kuku, Mustafa Culha

**Affiliations:** Department of Genetics and Bioengineering, Faculty of Engineering, Yeditepe University, Atasehir, Istanbul 34755, Turkey; m.altunbek@gmail.com (M.A.); gamzeku@gmail.com (G.K.)

**Keywords:** gold nanoparticle, toxicity, cellular uptake, single-cell analysis, surface-enhanced Raman scattering

## Abstract

The need for new therapeutic approaches in the treatment of challenging diseases such as cancer, which often consists of a highly heterogeneous and complex population of cells, brought up the idea of analyzing single cells. The development of novel techniques to analyze single cells has been intensively studied to fully understand specific alternations inducing abnormalities in cellular function. One of the techniques used for single cell analysis is surface-enhanced Raman spectroscopy (SERS) in which a noble metal nanoparticle is used to enhance Raman scattering. Due to its low toxicity and biocompatibility, gold nanoparticles (AuNPs) are commonly preferred as SERS substrates in single cell analysis. The intracellular uptake, localization and toxicity issues of AuNPs are the critical points for interpretation of data since the obtained SERS signals originate from molecules in close vicinity to AuNPs that are taken up by the cells. In this review, the AuNP–living cell interactions, cellular uptake and toxicity of AuNPs in relation to their physicochemical properties, and surface-enhanced Raman scattering from single cells are discussed.

## 1. Introduction

**Figure 1:**
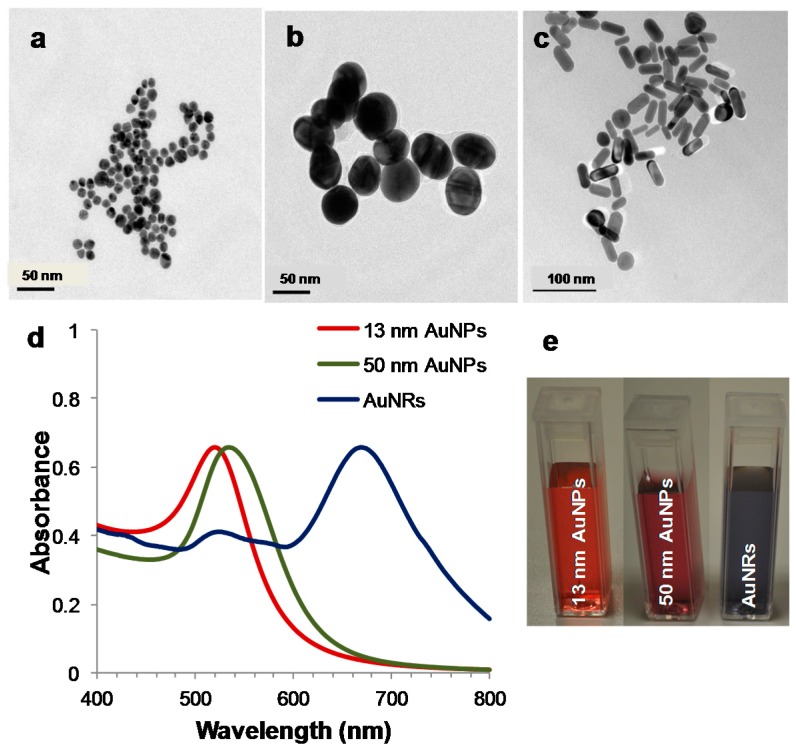
TEM images of: (**a**) 13 nm; and (**b**) 50 nm spherical (AuNPs); and (**c**) rod shaped (AuNRs) gold nanomaterials; (**d**) their UV/Visible spectra; and (**e**) images of corresponding colloidal suspensions (image courtesy of Nanobiotechnology laboratory at Yeditepe University).

Gold nanoparticles (AuNPs) are noble metal particles with tunable optical properties, which make them unique nanostructures in several applications including sensing, imaging and drug targeting. The optical property of the gold due to its strong interaction with electromagnetic radiation in the visible region of the spectrum makes it one of the unique noble metals. Upon interaction with light, it simultaneously absorbs and scatters at the same time. The absorbed light causes the enhanced oscillation of the metal’s electron system as the frequency of the absorbed light overlaps with the oscillation frequency of the electrons. As a result, an electromagnetic field called surface plasmons is formed on the nanostructured metal surface. While the absorbed light is transduced to heat by surface plasmons, the scattered light can be collected for imaging applications. The changes in size, shape, aggregation status and the composition of the particle as well as the dielectric constant of surrounding medium strongly influence the surface plasmon formation and the amount of light scattered. The surface plasmon resonance (SPR) wavelength can easily be monitored with UV/Visible spectroscopy. As representatives, Figure 1 shows transmission electron microscopy (TEM) images and UV/Visible spectra of spherical (13 nm and 50 nm) and rod shaped AuNPs prepared with citrate reduction and seed-mediated surfactant-assisted synthesis approach, respectively. The absorbance spectra demonstrate the influence of size and shape of the AuNPs on SPR. The increase in the size of spherical AuNPs shifts the SPR band to a longer wavelength. The rod shaped AuNPs have two absorption bands corresponding to the oscillation of electrons along with width and length of nanorod [1]. The interaction of noble metals with electromagnetic radiation is extensively studied and there are many excellent reviews and books available for readers [2,3,4]. Since it is out of the scope of this review, details of plasmonics are excluded here.

AuNPs used in in vitro cell studies are usually prepared in the size range of 2–100 nm mainly with wet-synthesis methods [5,6,7,8,9,10]. A reducing agent such as tri-sodium citrate and sodium borohydride is commonly employed. Since the goal is to use them in living cell studies, it is important to use a nontoxic reducing agent. For example, Cetyl trimethylammonium bromide (CTAB) is used to make rod shaped AuNPs but it is toxic for living cells [11,12,13]. AuNPs are not only used as-synthesized but also after surface modifications. The goal with surface modification is either to reduce the toxicity or to attach functional groups or coatings for targeting or delivery or both [14,15,16,17,18,19]. For minimal toxic effect on cells, surface chemistry, size and shape of the AuNPs as well as their uptake route should be carefully considered since AuNPs are allowed to interact with living cells by adding them into cell culture.

## 2. Cellular Interaction and Toxicity Concerns of Gold Nanoparticles

### 2.1. Cellular Interaction and Uptake of AuNPs

Apart from their size, shape and surface chemistry, which will be discussed in detail in the next section, the aggregation/agglomeration status, protein adsorption kinetics and incubation time of the NPs as well as the tested cell type and morphology are considered as factors influencing the toxicity of AuNPs. Basically, the AuNPs can adhere on cell membrane by either specifically or non-specifically through electrostatic interactions, Van der Waals’ forces, hydrophobic or hydrophilic forces and ligand–receptor binding, which initiates a sequence of dynamic interactions and kinetic processes as reviewed in detail by Bao et al. [20].

The possible internalization of NPs depends on energy-dependent endocytic transport mechanisms including phagocytosis, micropinocytosis, and clathrin- and caveolin-dependent pathways [21,22]. The phagocytic pathway [23,24] involves in the uptake of solid particles larger than 750 nm by specialized mammalian cells whereas pinocytosis [25] or macropinocytosis [26,27] enroll in the uptake of particles from a few to several hundred nm. The primary uptake mechanism for NPs is probably clathrin-mediated endocytosis, which form endocytic vesicles smaller than 100 nm to engulf the particles [28].

A further NP-uptake mechanism was also identified to be independent from clathrin-mediated mechanism, in which 50 to 80 nm size of plasma membrane vesicles containing cholesterol, sphingolipids and caveolae are formed [29]. The caveolae-mediated endocytotic uptake of AuNPs is mostly observed in endothelial cells [29,30].

The type of cells and their morphology also influence the NP uptake efficiency [31]. For example, it was found that the intracellular uptake of NPs by the cells of smaller size was low compared to the larger ones [31]. However, the NPs per unit area of cells in smaller cells were higher than larger cells since the cell surface tension due to cell/NP interaction in larger cells reduced the cellular uptake efficiency. The results also imply that the cell/NP contacting area has a positive role in cellular uptake, whereas induced cell membrane tension negatively influences the NP uptake efficiency.

### 2.2. Influence of Size, Shape and Surface Chemistry on AuNP Toxicity

Table 1 shows the summary of recent reports pointing out the effect of particle size and shapes of AuNPs on cellular uptake efficiency in different cell lines. Since we only focused on single-cell studies of SERS in this review, we only included the in vitro toxicity concerns of AuNPs. However, there are excellent reports and reviews about the in vivo toxicity studies in the literature [32,33,34,35,36].

When shape is considered, the spherical AuNPs are indicated as the most efficiently internalized AuNPs into cells [37]. The 50 nm particle size was found to be the most efficient size for cellular uptake [38,39]. In an in vitro study on model intestinal epithelial cell line (Caco-2), where a monolayer of Caco-2 cells were grown on a permeable support to mimic the absorption ability of gastrointestines, smaller particles (15 nm) were shown to be absorbed more rapidly whereas 100 nm size was found to accumulate in Caco-2 cells [39]. AuNPs of size 15 or 50 nm did not show any adverse effect on cells. However, the accumulated AuNPs with 100 nm size caused decrease in the mitochondrial membrane potential. Another study also showed similar results; 10 nm AuNPs were taken up rapidly while the intracellular accumulation of 25 nm AuNPs was higher [40].

The effective size was also tested among 10, 25 and 50 nm size of AuNPs loaded with 10-Hydroxycamptothecin (HCPT) for drug delivery [41]. Among them, 50 nm AuNPs loaded with HCPT were more effective to kill MDA-MB-231 cells due to the higher uptake efficiency.

The influence of particle size in relation to surface chemistry on AuNP–cell interaction and uptake was also demonstrated. A mechanistic study performed with 2, 4 and 6 nm spherical AuNPs modified with anionic, cationic and neutral ligands showed that the surface chemistry altered the internalization pathway in which the neutral NPs were taken up by passive diffusion [42]. The AuNPs with 2 and 4 nm sizes with anionic as well as cationic surface charge were internalized by endocytic pathway whereas 6 nm AuNPs used caveolae/lipid raft-mediated pathway. It was found that the rate of internalization was directly proportional to the cationic particle size; as the particle size increased, the intracellular uptake increased. However, it was inversely proportional with particles having neutral and anionic surface charges; as the particle size increased, the uptake efficiency decreased. It is clear that the surface chemistry has dramatic effect on the nature of AuNP–cell interactions and control over the uptake route compared to the size of AuNPs.

The uptake efficiency might also change depending on the tested cell type. For example, Stojiljković et al. evaluated the intracellular uptake by using citrate-reduced 15, 40 and 80 nm AuNPs on phagocytic neural (N9) and non-phagocytic (SH) microglial cells [43]. The 15 and 40 nm AuNPs were mostly internalized into the SH cells while 80 nm AuNPs were up-taken by N9 cells without inducing any cytotoxic effects.

The behavior of spherical AuNPs also changes in vitro and in vivo conditions since the NPs come across with different barriers in reticuloendothelial system (RES). The in vivo toxicity assessment of AuNPs with different sizes (3, 5, 8, 37, 50 and 100 nm) in mice showed that 8 and 37 nm AuNPs induced cytotoxic effect by causing physiological changes in lung, liver and spleen while others did not show a noticeable toxic effect [44].

As mentioned previously, AuNRs are commonly fabricated by seed-mediated growth method in the presence of cetyltrimethylammonium bromide (CTAB), which is highly toxic to living cells. The toxicity of CTAB-containing AuNRs can be reduced by modifying the surface with polyethylene glycol (PEG), phospholipids, silica shells, and proteins. Li et al. established a seedless, one-pot synthesis of CTAB-containing AuNRs by adding sodium oleate to the synthesis. This modification in the protocol also enabled them to obtain smaller sized AuNRs of about 7 nm diameter [45]. They compared the cytotoxicity of their 7 nm seedless-synthesized AuNRs (sAuNR-CTAB) to the 14 nm AuNRs obtained through seed-mediated synthesis (bAuNR-CTAB). The study was extended by modifying the particle surfaces either by surfactant exchange with oleate or bovine serum albumin (BSA) conjugation. Although both modifications improved the viability levels in the both cell lines used, BSA modification was slightly better probably due to receptor mediated endocytosis of BSA. Contradictory to common observations, sAuNRs were less cytotoxic than bAuNRs regardless of their surface modification even though their size was smaller. The addition of sodium oleate to the AuNR synthesis in their protocol can be considered as the main cause for this contradiction since the AuNR surface contains less CTAB. It was shown in a study by Tatini et al. that prolonged exposure of AuNRs in cell culture medium might lead to cytotoxicity even though the surface containing CTAB was initially coated with PEG [48]. Trace amounts of Ag, used in the synthesis of AuNRs, were also detected to be released from the AuNR surface which was considered as one of the causes of toxicity upon prolonged incubation.

In another study, CTAB-AuNR surface was modified with silica layer and further modified with folic acid (FA) to be effectively taken up by cancer cells [46]. The replacement of CTAB with a silica layer significantly decreased the cytotoxicity on HepG2 cancer cells. Further modification with FA provided efficient and rapid uptake by HepG2 cancer cells.

In order to utilize better heat and energy transfer efficiencies, different shapes of AuNPs are also investigated. For example, the intracellular uptake and cytotoxicity of nanocages (NCs) and AuNRs were compared on HUVEC and DU145 prostate cancer cells by Robinson et al. [47]. The cytotoxicity of both NPs was decreased by the removal of CTAB and PVP and coating with PEG on the surface of NCs and NRs. The internalization of both NCs and NRs in HUVEC cells were found to be higher than in DU145 cells, but the NCs taken up into DU145 cells was higher than nanorods. Another study comparing gold nanostar and nanosphere cytotoxicity showed that nanospheres decreased the cell viability compared to nanostars [37].

In summary, the toxicity of AuNPs depends on many factors that might synergistically affect the overall toxic response. Among them, the surface characteristics can be considered as the most contributing factor which is the first step of AuNPs–cell interaction. The literature also demonstrates that, in most cases, the toxicity of AuNPs can be bypassed through changing the surface chemistry. Therefore, the surface chemistry should carefully be considered and fine-tuned for precise targeting and avoiding damage to the cells.

## 3. Single-Cell Analysis and Surface-Enhanced Raman Scattering

### 3.1. Single-Cell Analysis

In multi-cellular biological systems, cells are specialized to perform specific tasks to organize and maintain the complex structure of the system [49,50,51,52,53,54,55,56,57]. Therefore, heterogeneity among cell populations occurs even at the embryonic stage by differential expression at genomic, transcriptomic and proteomic levels. The importance of cell-population heterogeneity also appears in immune response-related mechanisms in the body and tumor-related issues such as tumor formation, metastasis, diagnosis and therapy, all of which comprise a high percentage of hot topics in biomedical research [50,58,59,60,61,62,63]. The identification of individual cells based on their morphological or biochemical properties plays a critical role for the development of new therapeutic approaches by targeting the distinct properties of cancerous and metastatic cells [50,58,59]. In addition, the discrimination of the cell properties that cause pathogen infection in a population can shorten the therapeutic processes [64]. The analysis of single cells during the embryonic development is also important in early stage detection for the chromosomal aneuploidy [65].

**Figure 2:**
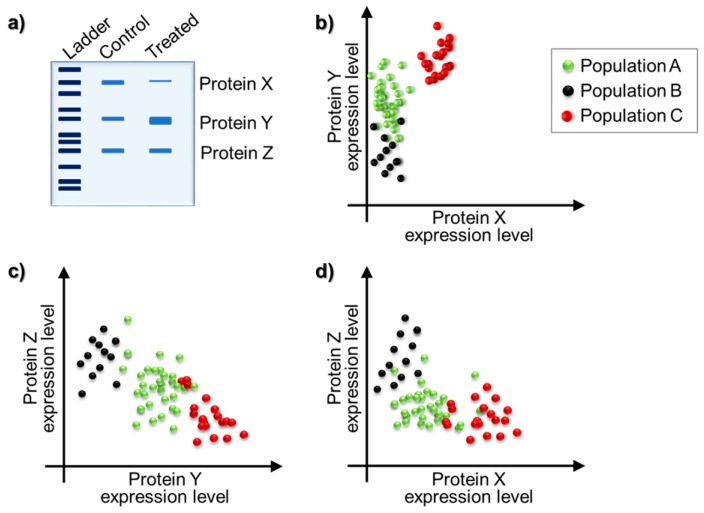
(**a**) Protein expression analysis results of a drug-treated and non-treated tumor model; and (**b**–**d**) scatter plots of protein X, Y and Z expression levels in cell populations A, B and C. Dots represents single cells (image courtesy of Nanobiotechnology laboratory at Yeditepe University).

Despite the extensive amount of information gathered from in vitro studies on the mentioned fields, our knowledge is still lagging behind in terms of detailed explanation of these processes. For instance, in patients receiving cancer therapy, the tumors often develop resistance to a very promising drug that is targeting a specific cancer type. To understand the reason behind drug resistance, utilizing analysis tools based on bulk amount of cells might be misleading. In Figure 2, a hypothetical protein-expression analysis was given to visualize the importance of single-cell analysis in heterogeneous cell populations. In this hypothetical example, imagine a tumor consisting of three cell populations; A, B and C, all of which might originate from a different cell type, thus carry distinct protein expression characteristics. Prior to treatment, termed “control group” here, the proteins X, Y and Z might be three hypothetical proteins that were equally expressed. Upon treatment, protein X was seen to be down-regulated, protein Y was up-regulated whereas protein Z levels were seen unchanged. The data obtained from the gel image show the overall protein expression amounts within hundreds of cells. However, what is seen might not be what is actually going on among the three cell populations. Population C might have started to overexpress protein X, whereas populations A and B have lowered the expression levels. Similar cases might occur for other proteins and populations. Taken together, the combination of these fluctuations in protein expression results in what we see as “drug resistance”.

Several techniques have been applied for single-cell studies including microscopic imaging [66,67], patch-clamp [68,69], comet assay [70,71], flow cytometry [72,73], mass spectrometry [74,75], electrophoresis [70,76], fiber optics [77,78] and surface-enhanced Raman spectroscopy (SERS) [15,19,79,80]. Some of the conventional approaches to analyze living cells are based on their surface markers [81,82,83], secreted proteins [84,85,86], metabolites [87,88], phosphoprotein signaling pathway elements [89,90], genomes [91,92] and transcriptome [93,94]. For example, flow cytometry equipped with multiple lasers and detectors can analyze 12 different surface protein markers by multicolor labeling [95]. Due to generation of multiple data from the single cells, the computational tools are also combined with experimental studies for the corresponding quantification between the cells [96]. Further advances allowed the analysis of cells without destruction, which can be separated and re-used for further studies [97]. The advances in the analysis of single cells still continue by the improvement of many techniques generating multiple data.

With the advances in photonics and electronics, several novel approaches were also reported in the literature [59,98]. For example, atomic force microscopy (AFM) was comparatively used to study the elasticity of healthy and diseased cells [99,100]. In another approach, tip-enhanced Raman spectroscopy (TERS) was used to study cell surface structures [101,102]. In TERS, an AFM tip coated with gold is used for the signal enhancement. The tip of the cantilever and impinging laser light coincides at the same location on the cell surface where both chemical and topographic information is collected. In another approach, the conductivity of cells is used to analyze the cellular response [103,104]. A label-free protein foci formation analysis on oxidatively stressed HeLa cells was recently accomplished by utilizing infrared nanospectroscopy [105]. In most of the above-mentioned approaches, one property of a cell or information from a certain location of the cell is obtained at a time.

### 3.2. Raman Spectroscopy and Surface-Enhanced Raman Scattering

Raman spectroscopy (RS) is one of the techniques employed for the analysis of single cells [106,107,108,109]. RS measures the inelastically scattered photons produced by vibrational frequencies from a molecule or a molecular structure with the excitation of a monochromatic light, mostly a laser [110]. The technique provides fingerprint information about the bond structure of a molecule. Therefore, it can be used to identify components of a molecular mixture. It is a non-destructive and non-invasive technique and may provide valuable molecular information from a sample almost without sample preparation. Unlike IR spectroscopy, the influence of water is limited due to the very weak scattering originating from water. A Raman spectrum from single cell can provide information about the nucleic acids, carbohydrates, proteins and lipids inside a cell [111]. The technique is very convenient to study a single cell in its natural environment without any fixation or labeling step prior to analysis, which may cause changes in cellular processes [112,113,114]. A laser line towards the IR region of the spectrum can conveniently be used to reduce of photo-induced damage to cells and fluorescence background originating from cellular matrix. For example, most of the cell analysis performed with a 785 nm wavelength laser showed no damage to cells although high laser power with longer acquisition time was applied [113,114]. Although RS spectroscopy has several unique features to study a large variety of materials, it suffers from very weak scattering of photons. In early 1970s, it was discovered that when a molecule is brought close enough to a noble metal surface such as gold or silver, Raman scattering was enormously enhanced [115,116]. The enhancement mechanism is now explained with the chemical [117] and electromagnetic components [118]. The chemical enhancement is considered to have lesser effect than electromagnetic since it is limited by the affinity of molecule to the noble metal surface [119,120].

### 3.3. SERS Substrates Used in Biological Applications

Since the discovery of SERS, it has been used in numerous applications [121,122,123]. The most preferred SERS substrates are gold and silver due to the fact that the electron system of these metals can be excited in the visible region of the spectrum [3]. The better SERS enhancement with silver nanoparticles (AgNPs) is reported but they are not very suitable for living single-cell SERS studies for their toxicity concerns and oxidation [124] even though there are examples of their use in single cell studies [101,125,126]. AuNPs are more suitable for single-cell SERS studies due to their biocompatibility, stability and synthesis of particles with controllable size and narrow size distribution [127].

The size of the NPs plays an important role for the enhancement efficiency of SERS. For example, the Ag and Au NPs with particle sizes between 20 and 74 nm provide more effective enhancement [119,120,127,128,129]. In addition, the extraordinary SERS enhancement is observed from the “hot spots”, which are formed at the junctions of aggregated NPs [127]. The aggregation causes the shift of AuNP SPR band towards longer wavelengths, which may allow to use a longer wavelength laser to reduce the radiation damage to the cells [130].

In a recent study, different sizes of gold nanorods (AuNRs) were used to demonstrate their effects on SERS [131]. It was found that although the AuNRs had similar SPR bands, the scattering intensity increased and the SPR band was broadened, as the particle size increased. However, the increased size decreased the SERS intensity, which was explained by the faster plasmon damping time in large nanoparticles and weaker radiation damping with small particles. This study also demonstrated that the SERS enhancement was responsive to the change in the excitation wavelength; smaller particles showed higher enhancement with the longer wavelength excitation.

The various shapes of AuNPs have been investigated to verify the effect on SERS. Nanorods, nanostars, nanoflowers have already been investigated in order to obtain reproducible SERS spectra of biological molecules with better enhancement in a cell-free environment [131,132,133,134]. For example, gold nanostars and nanoflowers provide better enhancement than spheres since the rough surface provides better electromagnetic enhancement and also provides a large surface area for the molecule adsorption [135,136]. In another study, the comparison of rhodamine-6G (R6G) SERS spectra obtained with star, triangle and sphere shaped AuNPs showed that the characteristic Raman bands of R6G at 613, 775, 1130, 1278, 1364, 1389, 1512, and 1651 cm^−1^ were able to be observed with nanostar shape demonstrating the influence of the shape on enhancement of the SERS spectra [132].

The researchers also focused on the synthesis of the NPs with different compositions to obtain biocompatible SERS substrates providing reproducible spectra with better enhancement. For example, Ag_core_/Au_shell_ NPs were synthesized to utilize SERS enhancement of Ag core and to make the SERS substrate biocompatible for biological applications by including the Au shell [137].

### 3.4. SERS-Based Single-Cell Studies

A single cell has a dynamic and complex heterogeneous intracellular environment consisting of DNA, RNA, proteins and lipids within several intracellular compartments. The discrimination of the complex molecular structures is achieved by either sending bare SERS substrates or targeting the cells with the SERS substrates containing selective molecules such as anti-epidermal growth factor receptor, herceptin proteins, nuclear localization and cell-penetrating peptides. SERS arises from the analytes themselves or the changes in recognition molecules such as antibodies and aptamers that are attached on substrate [16,114]. After AuNPs are internalized into cells, they might accumulate in the sub-endosomal compartments at different concentrations depending on their size and surface chemistry, unless they carry organelle-targeting moieties [138,139,140,141]. Therefore, it is important to understand the localization of AuNPs for the interpretation of SERS spectra.

The very first report on single-cell SERS analysis on living cells dates back to 1991 by Nabiev et al. even though AgNPs were used as SERS substrates in the study [142]. It was possible to identify the difference in the interaction of doxorubicin with the cytoplasm and the nucleus. Almost a decade after this first report, in 2002, AuNPs were utilized as SERS substrates in single living-cell SERS by Kneipp and co-workers [143]. Then, until 2010, studies focused on various possible uses of AuNPs in single-cell SERS. In 2004, intracellular pH [144]; in 2005, the use of multivariate statistical tools to differentiate an extrinsic molecule in the intracellular compartments [145]; in 2007 anti-EGFR conjugated AuNRs as cancer cell diagnostics tool [146] as well as intracellularly grown AuNPs as SERS substrates [147]; in 2008, intracellular quantification of an exogenous chemical [148] and in 2009, traces of cellular dynamics during AuNP transport were reported for the first time [149]. Current research in single-cell SERS mostly focus on high-throughput analysis, multiplexed imaging of various biomarkers, theranostic applications and time-resolved cellular dynamic changes [150].

Without organelle-specific targeting, it is possible to obtain overall cellular response signals by using bare AuNPs. In a recent study by Kuku et al., cytotoxic response of cells incubated with various nanomaterials were investigated via SERS by using 50 nm sized citrate-reduced spherical AuNPs as SERS substrates [151]. It was possible to plot cell-type and nanomaterial dose-dependent responses in the SERS spectra.

In a proof-of-concept study by Xu et al. the AuNP-AuNR self-assembled structures were investigated for a possible detection of small molecules such as nitric oxide, glucose, polyamines, NADPH/NADP^+^ in HeLa cells [152]. The authors suggested that the proposed approach could detect the small molecules in a living cell simultaneously. They point out that the AuNP-AuNR nanostructures can be an alternative to the detection of small molecules that an immunoassay based approach is not available.

SERS based monitoring of the intracellular pH changes which can be helpful to observe cellular functions such as endosomal trafficking, cell membrane polarity, metabolism, growth and proliferation was also reported. Jaworska et al. modified the surface of spherical AuNPs with pH responsive 4-mercaptobenzoic acid (MBA) [153]. The acidic pH caused increase in the SERS signal at 1720 cm^−1^ corresponding to symmetric stretching of the COO- group while the neutral and alkaline environments caused deprotonation of COO- group increased the stretching vibration of C=O bond intensity at 1400 cm^−1^ and a shift to 1420 cm^−1^ was observed in cell free environment. Comparing the ratios of I_1400–1420_/I_1070_ and I_1720_/I_1070_ versus pH values of Henderson–Hasselbalch plots showed a linear response to pH changes. The MBA-AuNPs induced any adverse effect neither on cell morphology nor cell viability. The obtained SERS signal originated only from the MBA molecules after AuNPs-MBA internalized into the cells. The SERS spectra enabled to monitor the changed pH from 4 to 9 as the MBA-AuNPs were transferred through the endocytic compartments. Due to intracellular stability of MBA-AuNPs probe, they can be good candidates of environmental or pathological stimuli monitoring using SERS. 

SERS tags that are decorated with cell-surface biomarkers are seen as alternatives to the existing fluorescence-based tags, which suffer from photobleaching, spectral overlap of multiple dyes and chemical instability. For example, a SERS tag was prepared with coating of AuNPs (15 nm) core with a poly adenine to form a uniform nanogap of ~1 nm which as then decorated with strong Raman reporters 4,4′-dipyridyl (44DP), and 5,5′-dithiobis(2-nitrobenzoicacid) (DTNB) [79]. The Au core was then coated with different thicknesses of Au shell, resulting in NPs ranging from 40 to 100 nm diameter (Au@Au core–shell). Among these Au@Au core–shell NPs, the most enhanced signal was observed with 76 nm sized ones. The selected 76 nm Au@Au core–shell NPs were then functionalized with hyaluronic acid (HA) to make them bind to over-expressed HA receptor biomarker, CD44 protein, on the cell surface of human colon carcinoma cells. The study demonstrated the possibility of multiplexed single-cell biomarker imaging and early detection of cancer.

Monitoring the mitochondria can also provide information about the apoptosis, ROS production and allow the development of therapeutic strategies for the disease models. SERS substrates can be directed through the mitochondria by modifying the surface with mitochondria localization signal (MLS) or delocalized lipophilic cations ligands [154,155]. Kang et al. developed AuNP-DNA core Au shell NPs as substrates providing higher SERS enhancement when excited in the near infrared (NIR) region [17]. Further modification with mPEG thiol, RGD peptide and MLS peptide (MLALLGWWWFFSRKKC) enabled mitochondrial targeting. Treatment with potassium cyanide (KCN) inducing cell death after localization of NPs into the mitochondria showed the spectral changes over time. The same study also demonstrated the efficiency of AuNP-DNA core-Au shell NP substrates to monitor the cytoplasm and nucleus with specific functionalization.

Among the other compartments, nucleus is the most important compartment of the cells, where the cellular functions are directed. It is enclosed by a double membrane with 9 nm diameter pores [156]. The transportation of AuNPs with larger diameters than 9 nm into the nucleus is possible by attaching nuclear localization signal (NLS) peptides, which can be achieved by attaching a cysteine residue to the C-terminus of NLS [15,157,158,159,160,161,162]. Huefner et al. monitored the differentiation of neuroblastoma cells using SERS by targeting 40 nm AuNPs modified with a cysteine attached NLS peptide, CGTGPKKKRKVGGK. The differentiation was explored by increase in SERS peak intensities of proteins and DNA/RNA ratio [160]. In another study, the 25 nm AuNPs modified with NLS and Arginylglycylaspartic acid (RGD) peptides were used to monitor the efficiency of two widely-used anti-cancer drugs, cisplatin and 5-fluorouracil [161]. The increased intensity of the Raman shifts at 1000 and 1585 cm^−1^ that were attributed to the phenylalanine and DNA bases were identified as the cell death bands in SERS spectra. El-Sayed and co-workers also demonstrated that the increased localization of AuNP clusters in nucleus was achieved by increasing the NLS-peptide on AuNP surface [162]. Using the NLS peptide-modified AuNPs, they also monitored the molecular changes in the nucleus corresponding to the cell death that was induced by photothermal therapy in real time SERS [58].

Another AuNP-delivery approach bypassing the endolysosomal pathway as well as the targeting moieties was to use an optimized electroporation method. In this method, Au-Ag core–shell NPs with a Raman reporter, 4-MBA, on the gold core were delivered to CA46, Burkitt’s lymphoma cells. Similar to the small-molecule detection study reported by Xu et al., it was possible to detect and plot the distribution of lipids and phenylalanine in the cytoplasm [152,163].

As highlighted above, the SERS spectra of the single cells highly depend on the uptake and aggregation of AuNPs in cellular compartments. In each type of cells, the formation of similar environment around AuNPs and their aggregates for SERS might not be possible due to different intracellular uptake efficiency of AuNPs. El-Said et al. designed a sensitive SERS-active substrate surface by placing ordered gold nano-dots on indium tin oxide (ITO) surface to overcome the problems associated with low reproducibility and high variability depending on intracellular uptake of AuNPs [80]. The gold-nano dot SERS substrate enabled monitoring cell differentiation, cell cycle stages and live/dead cells without influencing the viability of attached cells with any sample preparation step. The substrates can provide a continuous system for time-dependent monitoring such as drug discovery studies.

## 4. Conclusions

In this report, we aimed to review the single-cell analysis with SERS by stressing the interaction of living cells with AuNPs, which are commonly used SERS substrates. Since the observed SERS spectra originate from molecules and molecular structures on AuNP surfaces, their intracellular uptake and toxicity are also included in the review. The size, shape, aggregation status, and surface chemistry of the AuNPs not only determine their interaction with cells and their intracellular localization but also affect the spectral pattern on the SERS spectra as a result of molecular events in a living cell. Most of the single-cell SERS studies utilize AuNPs of around 50 nm diameter sizes, which were shown to result with the least toxic as compared to smaller or larger AuNPs than this size. In addition to the toxicity concerns, the size of the AuNPs is a parameter for sufficient SERS activity. In order to collect a SERS spectrum with high sensitivity, the average size of the AuNPs should be 50 nm. Coincidently, the AuNPs with 50 nm of average size qualify for both minimal cytotoxicity and high sensitivity. It is important to note that a tolerable AuNP concentration should be used to prevent their influence on the cellular processes.

A selectivity element such as an antibody or aptamer can be introduced to the surface of the AuNPs. The current literature indicates a trend that SERS tags decorated with antibodies for selectivity will enable multiplexed theranostic applications when combined with flow cytometry. Such applications are seen as alternatives to the existing fluorescence-based ones, especially due to their superior resistance to photobleaching. Organelle-targeting AuNPs, on the other hand, have been shown to provide valuable information on the monitoring of organelle-specific changes. It is important to note that in live-cell analysis the amount of AuNPs aggregated around the organelle of interest might influence the cellular integrity. Therefore, in such studies, the amount of AuNPs should be optimized to obtain sufficient SERS signals without adverse effects on cells.

Although SERS based approach is an exciting idea for single cell analysis, there are also some challenges to overcome such reproducibility and quantitation. Due to the nature of the technique, there are many parameters influencing the reproducibility of SERS measurement from a single cell: size, shape, aggregation status of AuNPs in the cell and laser frequency should be carefully evaluated to assure the observed changes on the SERS spectra due to the cellular processes. The quantitation of the events obtained from SERS spectra is another challenge. As mentioned earlier, the aggregation status of the AuNPs highly influences the intensity of bands on SERS spectra. Therefore, unlike Raman spectroscopy, quantitative analysis of observed SERS bands is not highly accurate even though semi-quantitative information can be extracted.

Finally, a general challenge in single-cell research regardless of the applied technique is the processing of large data sets. Improved algorithms for data reduction have been utilized to overcome this challenge. However, more studies are required to establish routine analysis of single-cells by using SERS.

In summary, SERS based single cell analysis is slowly evolving to be an important approach to study cells. Although the challenges are ahead, studies up to date are promising and the approach has the potential of contributing tremendously to the single cell analysis.

## Figures and Tables

**Table 1:** Summary of influence of AuNP size, shape and surface chemistry on cellular uptake efficiency.

AuNPs Size/Shape	Surface Chemistry	Cell Line	Uptake Efficiency	Ref.
15, 50 and 100 nm spherical	Tri sodium Citrate	Intestinal epithelial (Caco-2) cells	Uptake and spread of 15 nm size was more rapid. Best uptake efficiency was observed with 50 nm size whereas 100 nm size tended to accumulate.	[39]
10 and 25 nm spherical	Bovine Serum Albumin	Human cervical (HeLa) cancer cells	10 nm was taken up more rapidly than 25 nm. 25 nm accumulated in higher concentrations had better retention.	[40]
10, 25, and 50 nm spherical	10-Hydroxy camptothecin (HCPT)	Human breast cancer (MDA-MB-23) cells	The 50 nm HCPT-loaded AuNPs had unique advantages over smaller NPs in terms of killing MDA-MB-231 cells due to the higher uptake efficiency	[41]
2, 4, and 6 nm spherical	Cationic, neutral and anionic	Human cervical (HeLa) cells	The increasing particle size resulted in increased uptake for cationic nanoparticles whereas for neutral and anionic particles it decreased uptake efficiency.	[42]
15, 40 and 80 nm spherical	Tri sodium Citrate	N9 phagocytic microglial cells, Nonphagocytic neural SH cells	SH cells engulfed small particles between 15 and 40 nm in diameter while the larger particles with a diameter of 80 nm preferentially internalized into N9 phagocytic microglial cells.	[43]
7 and 14 nm diameter Rod shape	CTAB, oleate or BSA	Hematopoietic stem cell (HSC) and Human liver cancer (HepG2) cells	7 nm gold nanorods showed higher cell uptake compared to 14 nm independent of the surface modification.	[45]
40 nm diameter Rod shape	Silica coated Silica coated-folic acid modified	Human liver cancer (HepG2) cells	The cellular uptake of AuNRs@SiO_2_-FA was rapid while unmodified AuNRs@SiO_2_ showed no obvious binding or internalization.	[46]
Nanocages and Nanorods.	PEG coated	Human umbilical vein endothelial (HUVEC) and DU145 prostate cancer cells	Both nanocages and nanorods were taken up by HUVEC more than DU145 cells. The internalization of nanocages into DU145 cells was higher than nanorods.	[47]
34 nm Nano stars and 61 nm Nanospheres	HEPES (Nanostar) and Citrate (Nanospheres)	Fibroblast cells and microvascular endothelial (RFPECs) cells	Nanospheres have higher toxicity compared to nanostars.	[37]
